# Baby-led weaning, an overview of the new approach to food introduction: integrative literature review

**DOI:** 10.1590/1984-0462/2022/40/2020507

**Published:** 2021-08-23

**Authors:** Viviane Laudelino Vieira, Gill Rapley

**Affiliations:** a Universidade de São Paulo, School of Public Health, Centro de Saúde Escola Geraldo de Paula Souza. São Paulo, SP, Brazil.; b Canterbury Christ Church University, School of Public Health, Midwifery and Social Work, Canterbury, United Kingdom.

São Paulo, December 1^st^ of 2020.

Dear editors of Revista Paulista de Pediatria,

As researchers in the field of infant feeding, especially dedicated to the approach known as baby-led weaning (BLW), we consider the growing number of publications on the subject to be very positive. Particularly in Brazil, little has been produced regarding the possibility of babies leading the process of the introduction of new foods. Meanwhile, more and more families are looking for this option.

We have had access to the text published in December 2019 by Gomez et al.,^1^ entitled “Baby-led weaning, an overview of the new approach on food introduction: an integrative literature review”. We are pleased to highlight the attention given to the BLW method, but, at the same time, concerned with some of the statements put forward by the authors, which do not accurately reflect the literature. For this reason, we should like to take this opportunity to challenge the comments with which we disagree, with reference to the existing literature.

Let us start with the excerpt in which it is stated that: “Wright et al.^2^ reported that the children who followed the BLW method would search for food to feed themselves only at eight months, which generates a nutritional concern and suggests a less rigid approach in the first weeks”.

Wright et al.^2^ did not analyze babies submitted to the BLW method. They studied babies from a cohort study conducted in 1999-2000, a period that predates the first publications on the subject. These authors claimed that: “Most infants in the cohort started reaching out for food and eating finger foods between the ages of 4 and 7 months.” However, they also pointed out that: “In this cohort, there was a substantial discrepancy between an apparent capacity to self-feed and being given the opportunity to do so”. Therefore, as Wright et al.^2^ claim, the opportunity for self-feeding was not routine for most of the babies. Thus, they were not fed using the BLW approach. In fact, Wright et al.^2^ concluded that caution must be taken for babies with developmental delay, but that the BLW method would probably be viable for most children.

Now, let us comment on the following excerpt: “Cameron et al.^3^ compared two groups. A group of babies who practiced BLW in full and a group of babies who practiced Baby-Led Introduction to Solids (BLISS), considered an adaptation of BLW in the face of concerns about ingestion of micro and macronutrients and choking. There were no statistical differences, but the group exposed to the BLISS method was offered less food with a high risk of choking (3.24 versus 0.17 serve/day; p=0.027). These data corroborate the findings of Morison et al.^4^ and Fangupo et al. ^5^”

The study cited, by Cameron et al.,^3^ was a pilot study that used the following sample: BLISS (n=14) and BLW (n=9). It did not include a group of babies exposed to the traditional approach, that is, who were fed by a caregiver using pureed food. Therefore, one cannot say that the data corroborate the findings of Morrison et al.^4^ or Fangupo et al.,^5^ since these studies compared a group submitted to the BLISS method with another exposed to the traditional approach.

The authors use the text by Fangupo et al.^5^ to ratify the increased risk of choking in the BLW method. However, the original document states that: “there were no significant differences between groups as to the number of choking events at any time”. In the aforementioned work, a greater number of occurrences of the gag reflex was found, an event that does not constitute choking, and the authors themselves make this distinction.

The study mentioned in the previous paragraph, conducted by Brown^6^ with 1,151 mothers, reported that the “baby-led weaning was not associated with increased risk of choking and the highest frequency of choking on finger foods occurred with children who were given finger foods the least often” (excerpt taken from the original); that is, the increased risk of choking would be linked to babies who did not practice the BLW method (who would eventually eat with their own hands).

Thus, we address the editors of Revista Paulista de Pediatria expecting that the observations brought up here can be made accessible to the joournal’s audience, to prevent the training and updating of students, professionals and researchers from taking place on the basis of mistaken information.

At your disposal.

## References

[1] Gomez MS, Novaes APT, Silva JP, Guerra LM, Possobon RF (2020). Baby-led weaning, panorama da nova abordagem sobre introdução alimentar: revisão integrativa de literatura. Rev Paul Pediatr.

[2] Wright CM, Cameron K, Tsiaka M, Parkinson KN (2011). Is baby-led weaning feasible? When do babies first reach out for and eat finger foods?. Matern Child Nutr.

[3] Cameron SL, Taylor RW, Heath AL (2015). Development and pilot testing of Baby-Led Introduction to SolidS - a version of Baby-Led Weaning modified to address concerns about iron deficiency, growth faltering and choking. BMC Pediatr.

[4] Morison BJ, Taylor RW, Haszard JJ, Schramm CJ, Erickson LW, Fangupo LJ (2016). How different are baby-led weaning and conventional complementary feeding? A cross-sectional study of infants aged 6-8 months. BMJ Open.

[5] Fangupo LJ, Heath AL, Williams SM, Williams LW, Morison BJ, Fleming EA (2016). A Baby-Led approach to eating solids and risk of choking. Pediatrics.

[6] Brown A (2018). No difference in self-reported frequency of choking between infants introduced to solid foods using a baby-led weaning or traditional spoon-feeding approach. J Hum Nutr Diet.

